# Potentially suitable geographical area for *Colletotrichum acutatum* under current and future climatic scenarios based on optimized MaxEnt model

**DOI:** 10.3389/fmicb.2024.1463070

**Published:** 2024-09-23

**Authors:** Chun Fu, Yaqin Peng, Fengrong Yang, Zhipeng He, Habib Ali, Danping Xu

**Affiliations:** ^1^Key Laboratory of Sichuan Province for Bamboo Pests Control and Resource Development, Leshan Normal University, Leshan, China; ^2^College of Life Science, China West Normal University, Nanchong, China; ^3^Department of Agricultural Engineering, Khwaja Fareed University of Engineering and Information Technology, Rahim Yar Khan, Pakistan

**Keywords:** species distribution, habitat suitability, climate change, *Colletotrichum acutatum*, MaxEnt

## Abstract

Global climate warming has led to changes in the suitable habitats for fungi. *Colletotrichum acutatum*, a common fungus causing anthracnose disease, is widely distributed in southern China. Currently, research on the relationship between *C. acutatum* and environmental warming was limited. In this study, MaxEnt and ArcGIS software were used to predict the suitable habitats of *C. acutatum* under current and future climate conditions based on its occurrence records and environmental factors. The optimal MaxEnt model parameters were set as feature combination (FC) = lp and regularization multiplier (RM) = 2.6. Bio15, Bio12, Bio09, and Bio19 were identified as the main environmental factors influencing the distribution of *C. acutatum*. Under current climate conditions, *C. acutatum* was distributed across all continents globally, except Antarctica. In China, *C. acutatum* was primarily distributed south of the Qinling-Huaihe Line, with a total suitable area of 259.52 × 10^4^ km^2^. Under future climate conditions, the potential suitable habitat area for *C. acutatum* was expected to increase and spread towards inland China. The results of this study provided timely risk assessment for the distribution and spread of *C. acutatum* in China and offer scientific guidance for monitoring and timely controlled of its distribution areas.

## 1 Introduction

Anthracnose is a significant fungal disease that commonly occurs in plants and is mainly caused by species of the genus Colletotrichum (Cannon et al., 2012). Anthracnose often occurs on the leaves, stems, flowers, and fruits of plants. In the early stages of the disease, small spots typically appear on the infected areas, which later expand into dark brown circular lesions. In severe cases, the disease can cause the plant’s crown and stems to rot, leading to wilting and death of seedlings (Cannon et al., 2012). *Colletotrichum acutatum* is a common species within the genus Colletotrichum that can parasitize many plants and cause anthracnose. Initially, this fungus was isolated by researchers from diseased tissues of papaya, chili peppers, and delphiniums in Australia (Baroncelli et al., 2015). Nowadays, it is commonly found on tropical and subtropical crops such as strawberries, mangoes, citrus fruits, peach trees, avocados, bananas, coffee, and cereals (Thao et al., 2023). The conidia produced by *C. acutatum* are primarily spread through rainfall and wind (Dowling and Schnabel, 2020). When the conidia land on injured host plant tissue, they can cause secondary infections in the plant (Tan et al., 2022). The optimal conditions for *C. acutatum* to infect plants include high humidity, warm temperatures, wounds or natural openings on the plants, and susceptible or weakened plant varieties. The pathogen is most active in warm environments between 20°C and 30°C, where its spores rapidly germinate under high humidity or in the presence of water droplets on leaves. The spores then enter the plant tissue through wounds or stomata, leading to infection. When these conditions are present simultaneously, plants are more susceptible to infection by *C. acutatum*, resulting in severe disease outbreaks (Ruvishika Shehali et al., 2021). The high transmissibility and infectivity of *C. acutatum* make it a significant threat to the growth and yield of many fruit trees and crops, leading to decreased agricultural production and impacting the healthy development of the agricultural economy. Research has shown that there are both dominant and recessive resistance genes in *C. acutatum*. For example, the resistance of green pepper to the disease is controlled by a pair of recessive genes, while the resistance of mature fruit pepper is controlled by a pair of recessive genes (Mahasuk et al., 2009). In strawberry species, resistance to this fungus is a quantitative trait, with a dominant gene controlling high levels of resistance and a micro-effective gene controlling moderate levels of resistance (Mahasuk et al., 2009). Given that *C. acutatum* is sensitive to climate, global climate change could potentially expand its distribution. Therefore, understanding the current and future potential distribution of *C. acutatum* is crucial for preventing further infections and protecting crop growth.

Climate is a major factor influencing species distribution patterns. The Fifth Assessment Report (AR5) of the Intergovernmental Panel on Climate Change (IPCC) indicated that the annual mean temperature of the Earth’s surface had increased by 0.85°C over the past 130 years (1880–2012). While the mean temperature of the past 60 years (1951–2012) had increased by 0.72°C (IPCC, 2013). According to researchers’ projections of greenhouse gas emission scenarios, by the end of the 21st century, the global average surface temperature will increase by 0.3 to 4.8°C compared to current levels (Li et al., 2020). Climate change will lead to changes in ecosystems, directly impacting the survival of certain species and affecting the food chains they belong to, thereby further altering the composition of ecosystem patterns. Overall, global warming May accelerate the reproduction of heat-loving species and lead to the decline of cold-loving species (Parmesan and Yohe, 2003). Therefore, quantitative and visual analysis of climate factors affecting species distribution and the current and future potential distribution of species has become a focal point of biological research.

With the rise of statistical techniques and GIS tools, predictive models of species habitat distribution have rapidly emerged in biology and are widely used to forecast the potential distribution of invasive species, endangered species, harmful insects, and more (Gao et al., 2023). These models statistically correlate the geographical distribution of species with current environmental conditions, essentially being static and probabilistic in nature (Guisan and Zimmermann, 2000). Species distribution models (SDMs) encompass a wide range of disciplines, including ecology, biogeography, environmental science, conservation biology, species management, and more (Benavides Rios et al., 2024). Currently, many niche models are used to predict the potential distribution of species, including the bioclimate analysis and prediction system (BIOCLIM) (Ajene et al., 2024), the genetic algorithm for rule set production (GARP) (Townsend Peterson et al., 2007), the ecological niche factor analysis (ENFA) (Cushman et al., 2024), and the Maximum Entropy Model (Maxent) (Zhao et al., 2024). Species distribution models (SDMs) are widely used, but there are still some shortcomings. For example, the sample data for species occurrence May not always be accurate due to inaccuracies in recording or uncertainties in species classification, leading to potential biases in the samples (Elith and Leathwick, 2009). These niche models only consider the interaction between species distribution and environmental factors, lacking consideration for factors such as interactions between species, evolutionary processes within species, and disturbances from extreme conditions (Li et al., 2020). However, niche models remain a common tool for predicting species distributions because they can quickly infer the potential range of a species based on the presence of partial samples, thereby reducing the need for manual field surveys and re-recording processes.

The MaxEnt model, based on the principle of maximum entropy, is an effective and accurate tool for predicting species distributions. Maximum entropy principle serves as a criterion for learning probability models, where the model’s quality is assessed based on the magnitude of entropy; higher entropy indicates a better model (Steven et al., 2006). In general, the maximum entropy principle involves selecting the model with the highest entropy under specified constraints. The MaxEnt model utilizes current species occurrence data and various environmental factors such as soil, climate, and microbial factors to simulate both current and future potential distributions of species. It is known for its effective predictive capabilities, verifiable predictions, fast computation speed, and operational flexibility (Hirzel et al., 2006; Hou et al., 2023). In many studies, this model often operated using default parameters and all available environmental variables that can be collected. These operations increased the complexity of the model, reduced efficiency, and were not suitable for predicting suitable habitats for all species. Research had found that over-parameterized models tended to underestimate the availability of suitable habitats when transferring to new time periods, while under-parameterized models often overestimated it (Dan and Stephanie, 2011). In machine learning algorithms, complex models often led to overfitting, resulting in poor performance when predicting the spatiotemporal changes of species habitats (Zhao et al., 2022). Therefore, adjusting model parameters to reduce complexity and selecting appropriate environmental factors to enhance prediction accuracy was a necessary consideration.

In this study, after optimizing the parameters of the MaxEnt model and selecting environmental variables, the distribution changes of *C. acutatum* under different climatic conditions were demonstrated. The main objectives of this study were as follows: (1) Explore the suitable MaxEnt parameters and environmental factors for *C. acutatum*. (2) Investigate the environmental factors that significantly impact the distribution of *C. acutatum*. (3) Compare the distribution and changes of suitable habitats for *C. acutatum* under different time periods and economic emission scenarios. The significance of this study lay in providing scientific basis to prevent further spread of *C. acutatum*, manage anthracnose infections effectively, and enable relevant authorities to implement targeted measures, thereby reducing its impact on agricultural economies.

## 2 Materials and methods

### 2.1 Species occurrence data

The current distribution data of *C. acutatum* mainly came from the Global Biodiversity Information Facility (GBIF, https://www.gbif.org), supplemented with analysis of literature sources. Using Google Maps, latitude and longitude data were collected for 234 distribution points of *C. acutatum* where coordinates were not initially available. To reduce spatial autocorrelation (Marie-Josée, 1999), each grid (5 km × 5 km) was used as a standard, and ENM Tools 1.4 was employed to prune redundant data points, ensuring that each grid cell contains only one distribution point. The spatial resolution used was 2.5 arc-minutes (approximately 4.5 kilometers) (Gao et al., 2023). After pruning the coordinates using ENM Tools 1.4, the reduced dataset of coordinate points was imported into ArcGIS v10.8. In ArcGIS, data points that did not meet the criteria were removed, resulting in a final dataset of 164 distribution points.

### 2.2 Screening and processing of the environmental variables

**Figure 1:**
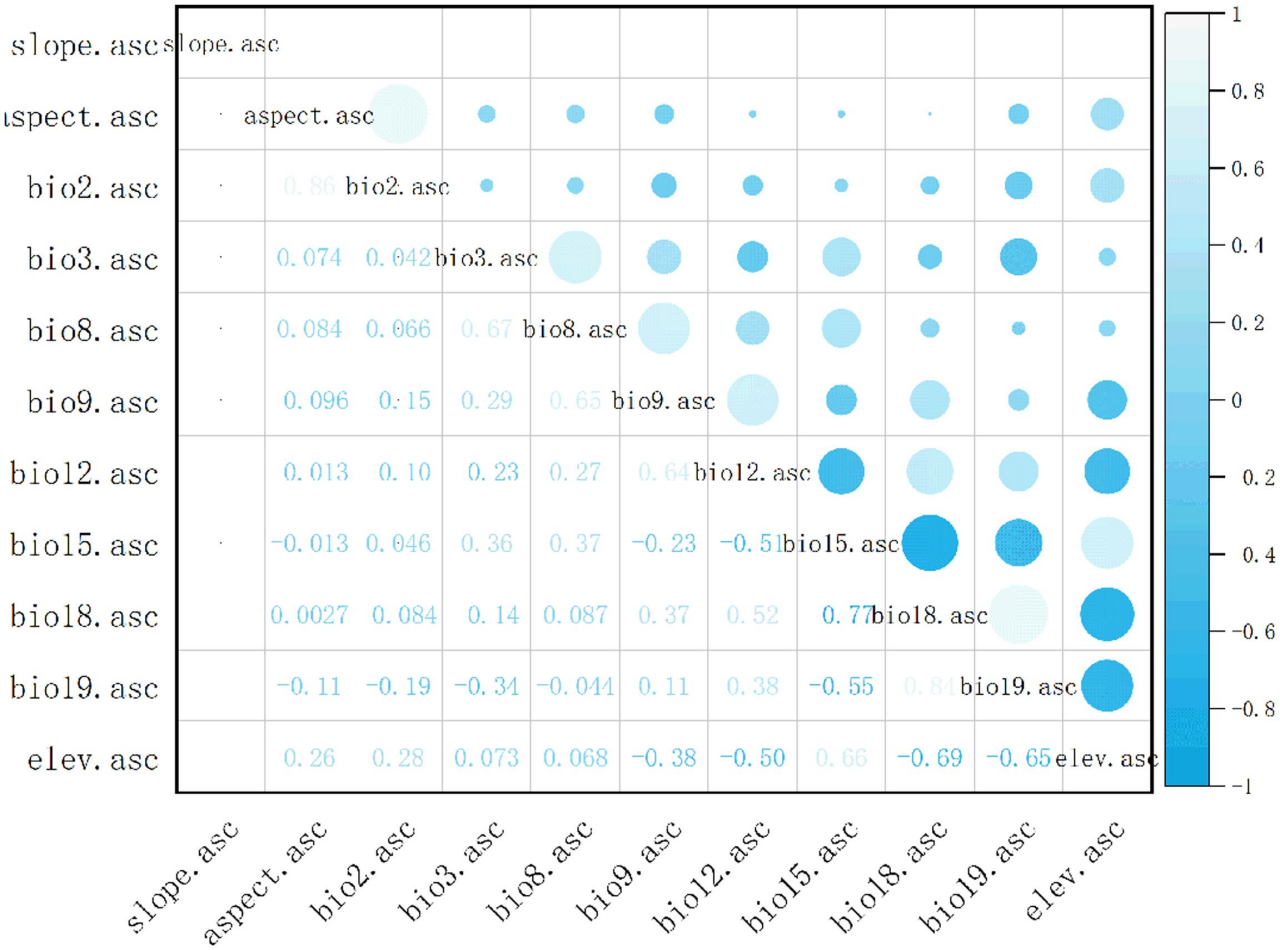
Correlation analysis of environmental variables.

Nineteen bioclimatic variables and 3 terrain variables were used to predict the current and future potential distribution of *C. acutatum* (Table 1). These variables were sourced from the World Climate Database^1^ and covered the time period from 1970 to 2000. They included spatial resolutions of 10 arc-minutes, 5 arc-minutes, 2.5 arc-minutes, and 30 arc-seconds, with modeling conducted at a resolution of 2.5 arc-minutes. Future climate data were obtained from the CMIP 6 (Coupled Model Intercomparison Project Phase 6) under the Beijing Climate Center Climate System Model (BCC-CSM 2-MR) climate model (Gao et al., 2023). In the study, scenarios from the 2050s (average from 2041 to 2060) and 2070s (average from 2061 to 2080) under SSP1-2.6, SSP3-7.0, and SSP5-8.5 were used for modeling. These scenarios represented different socioeconomic pathways and greenhouse gas emission levels that influenced the distribution of *C. acutatum*. Due to high correlations among these variables, directly using them in MaxEnt modeling could lead to overfitting. Initially, the environmental variables surrounding the distribution points of *C. acutatum* were analyzed using the sampling function in ArcGIS 10.8 software. Using SPSS software, a multicollinearity analysis was conducted to calculate the Variance Inflation Factor (VIF) (Ab Lah et al., 2021). This analysis preliminarily filtered out environmental factors with VIF values less than 100. VIF, also known as the reciprocal of tolerance, indicates severe multicollinearity when VIF > 100. Subsequently, ENMTools software was used to compute Pearson correlation coefficients (Figure 1), identifying and removing climate variables with correlation values greater than 0.8 to enhance the model’s accuracy (Wang et al., 2021). Nine environmental variables were retained: Slope, Elev (Elevation), Bio02, Bio03, Bio08, Bio09, Bio12, Bio15, and Bio19 (Table 2).

**Table 1:** Environmental variables related to the distributions.

Abbreviation	Climate variables	Unit
Bio01	Annual mean temperature	°C
Bio02	Mean diurnal range	°C
Bio03	Isothermality (bio2 / bio7) (× 100)	
Bio04	Temperature seasonality (standard deviation×100)	
Bio05	Max temperature of warmest month	°C
Bio06	Min temperature of coldest month	°C
Bio07	Temperature annual range (bio5–bio6)	°C
Bio08	Mean temperature of wettest quarter	°C
Bio09	Mean temperature of driest quarter	°C
Bio10	Mean temperature of warmest quarter	°C
Bio11	Mean temperature of coldest quarter	°C
Bio12	Annual precipitation	mm
Bio13	Precipitation of wettest month	mm
Bio14	Precipitation of driest month	mm
Bio15	Precipitation seasonality (Coefficient of variation)	
Bio16	Precipitation of wettest quarter	mm
Bio17	Precipitation of driest quarter	mm
Bio18	Precipitation of warmest quarter	mm
Bio19	Precipitation of coldest quarter	mm
Elev	Altitude (elevation above sea level) (m)	m
Slope	Slope	°
Aspect	Aspect	rad

**Table 2:** The nine environment variables used for modeling.

Variable	Environmental variables
Bio02 (°C)	Mean diurnal range (mean of monthly max temp-min temp)
Bio03 (°C)	Isothermality (Bio02/Bio07) × 100
Bio08 (°C)	Mean temperature of wettest quarter
Bio09 (°C)	Mean temperature of driest quarter
Bio12 (mm)	Annual precipitation
Bio15 (mm)	Precipitation seasonality (coefficient of variation)
Bio19 (mm)	Precipitation of coldest quarter
Elev (m)	Elevation
Slope (%)	Slope

### 2.3 MaxEnt model setting and selection

The MaxEnt model calculates the maximum entropy of species distribution by inputting current species occurrence data and environmental variables. This estimation helps to predict the suitable habitat for the species (Steven et al., 2006). The prediction results of the MaxEnt model are related to feature combinations (FC), regularization multiplier (RM), and maximum background points (BC). The model includes five feature types: linear (linear-L), quadratic (quadratic-Q), hinge (hinge-H), product (product-P), and threshold (threshold-T) (Wan et al., 2020). Combining these types of factors results in a total of 31 combinations. The regularization multiplier varies from 0.1 to 4 in increments of 0.1, yielding 40 values. Therefore, combining the feature combinations with the regularization multiplier results in 1240 parameter combinations. The default parameters of the MaxEnt model May not necessarily be suitable for predicting the potential distribution of all species. Muscarella et al. developed an R package called ENMeval to evaluate the combination of model parameters (Muscarella et al., 2014). Using R Studio, based on statistical significance (ROC with 500 iterations), predictive ability (omission rate, OR), and model complexity (AICc), the performance of these 1,240 candidate models was evaluated (Shi et al., 2023). The difference in Akaike Information Criterion corrected for small sample sizes (delta AICc) between the calibrated model and the current model is less than 2, and the omission rates are less than 5% (Zhao et al., 2022). Therefore, this calibrated model parameter combination is considered the optimal parameter combination. Finally, an optimized MaxEnt model was established. 75% of the presence data was randomly allocated as training data, while the remaining 25% was used as testing data. The feature combination included linear and product features, with a regularization multiplier of 2.6 and a maximum of 10,000 background points. The sampling method chosen was non-replacement subsampling, repeated ten times. The final MaxEnt prediction result was obtained by averaging the results of these ten runs. Model performance was evaluated using the Area Under the Receiver Operating Characteristic Curve (AUC and ROC). AUC values range from 0 to 1, with values closer to 1 indicating better predictive performance (Wang et al., 2020).

### 2.4 MaxEnt model analysis

The prediction results of the MaxEnt model are output in logistic format, where the species habitat suitability is quantitatively assessed on a scale from 0 to 1. Subsequently, these MaxEnt model predictions were imported into ArcGIS 10.8 software for reclassification and visualization. Using the Jenks’ natural break method, the habitat suitability of *C. acutatum* was classified into four categories: unsuitable (<0.08), low suitability (0.08–0.27), medium suitability (0.27–0.49), and high suitability (0.49–1) (Li et al., 2022). Finally, the ranges of each suitability zone were determined, and the areas of each suitability zone were calculated.

## 3 Result

### 3.1 MaxEnt model optimization and accuracy evaluation

**Figure 2:**
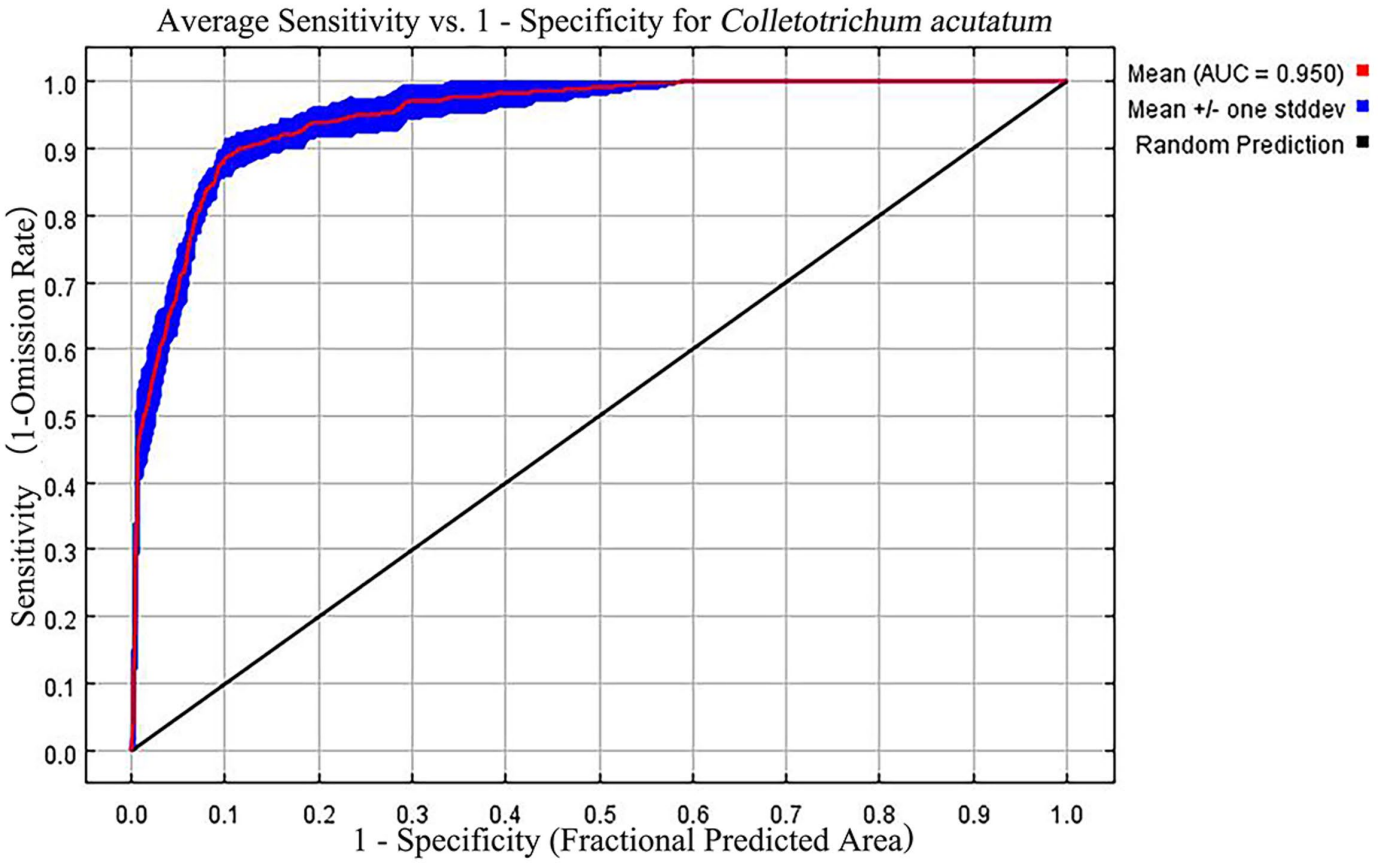
Receiver operating characteristic (ROC) curve generated by the MaxEnt model.

In this study, the optimized MaxEnt model showed excellent performance. The default parameters with FC = Auto feature and RM = 1 yielded an AUC value of 0.974 for the output results (Figure 2). After optimization, setting the MaxEnt model parameters to FC = lp and RM = 2.6 resulted in an AUC value of 0.950. The omission rate was 0.073%, and delta AICc was 0. The optimized MaxEnt model demonstrated high predictive performance, indicating that these optimized parameters can be used to predict the distribution of *C. acutatum* suitable habitats.

### 3.2 Relationships between the distribution of *Colletotrichum acutatum* and bioclimatic variables

**Figure 3:**
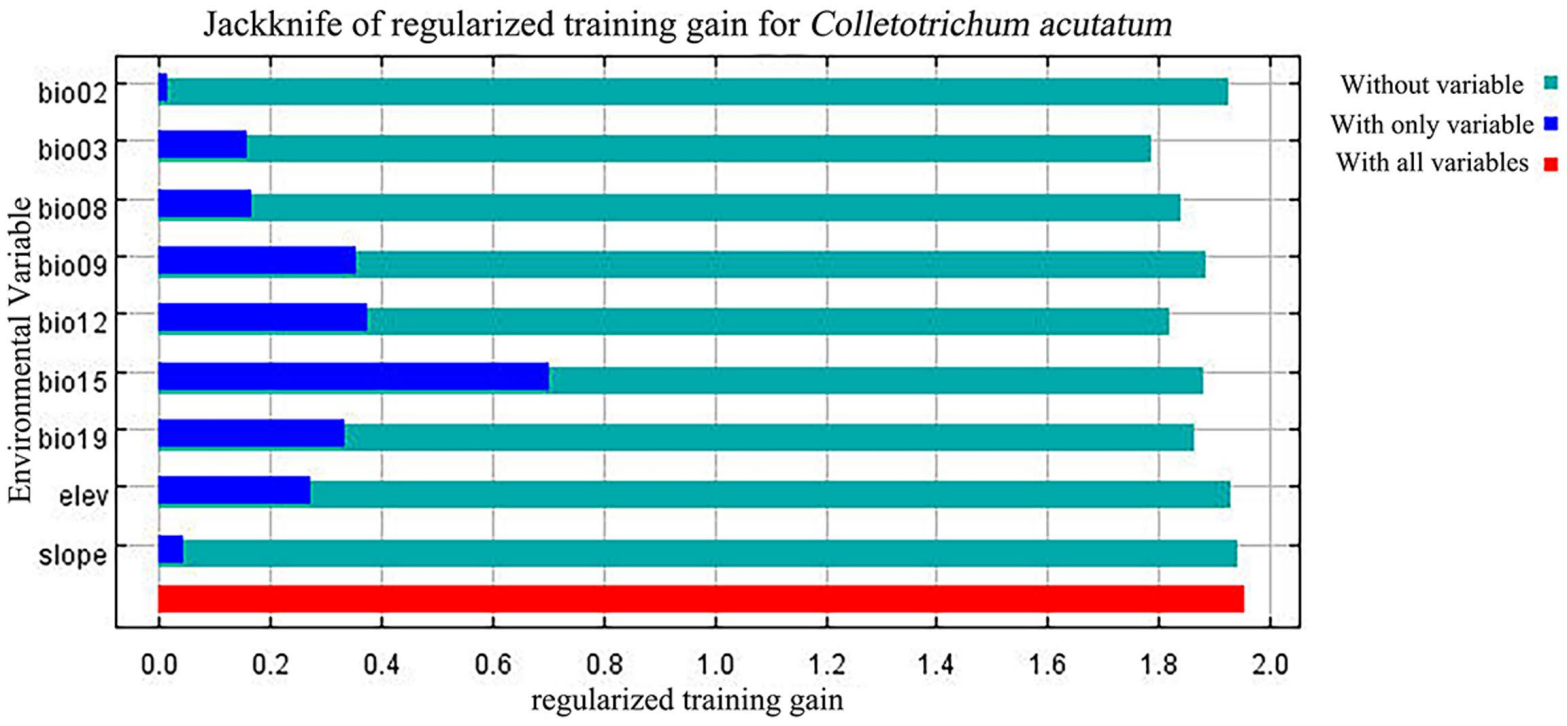
Jackknife test of variable importance for the MaxEnt model of *C. acutatum* distribution.

In these nine environmental variables, Bio15, Bio09, and Bio12 contributed significantly to the MaxEnt model predictions, with a cumulative contribution of 73.9% (Table 3). Among them, Bio15 had the highest contribution at 33.1%. The jackknife test (Figure 3) indicated that Bio15, Bio12, Bio09, and Bio19 had a substantial impact on the distribution of *C. acutatum*. Therefore, among the environmental factors, precipitation emerged as the primary factor influencing the survival of *C. acutatum*, with significant contributions from Bio15, Bio12, and Bio19.

**Table 3:** Contribution and permutation importance estimation of climate variables in the MaxEnt model of *Colletotrichum acutatum.*

Variable	Percent contribution (%)	Permutation importance (%)
Bio15	33.1	3.9
Bio09	21.4	24.6
Bio12	19.4	9.8
Bio03	9.9	18.5
Bio08	5.7	30.9
Elev	5.3	3.6
Bio19	3.0	5.8
Bio02	2.1	3.0
Slope	0.1	0.0

**Figure 4:**
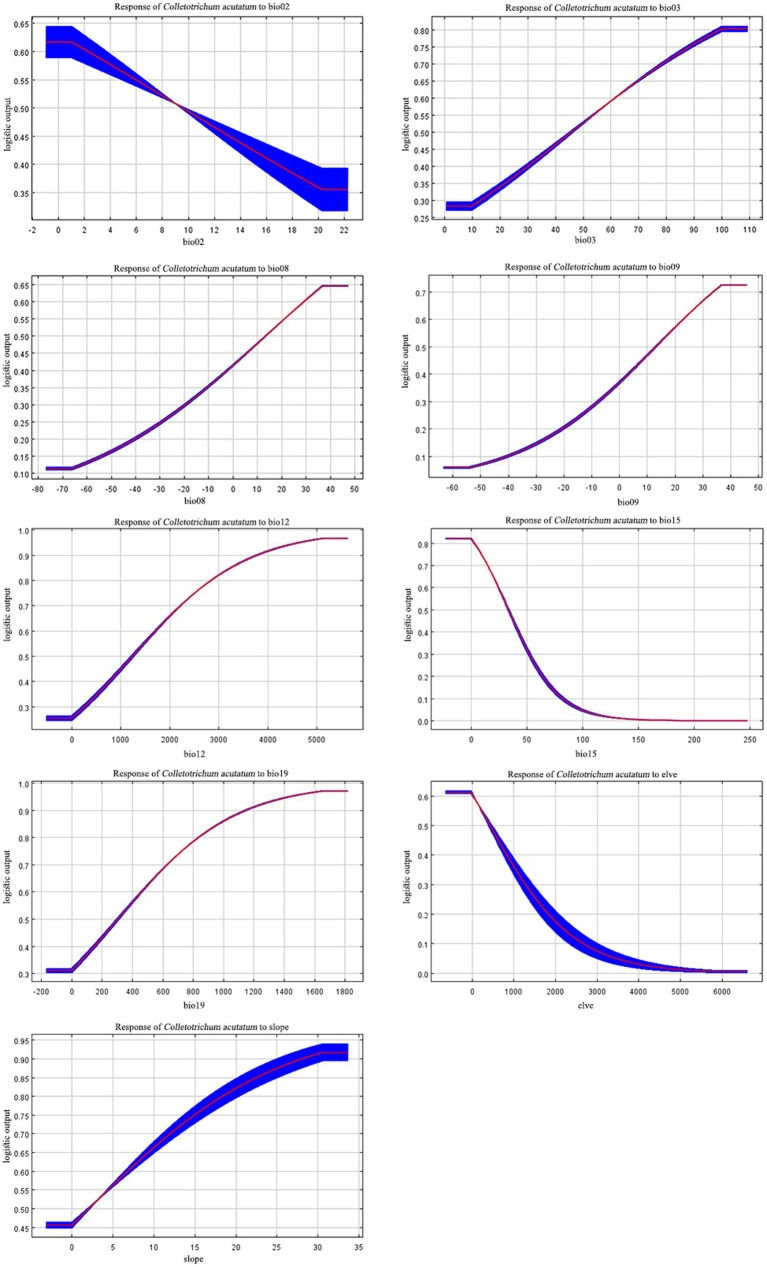
Response curves of nine environmental variables.

According to the response curves, areas suitable for *C. acutatum* survival exhibit seasonal variation in precipitation (Bio15) below 36.36 mm. Additionally, suitable environmental conditions for *C. acutatum* occurred when the precipitation of the coldest quarter (Bio19) exceeded 304.36 mm, annual precipitation (Bio12) exceeded 1283.76 mm, and the mean temperature of the driest quarter (Bio09) exceeded 11.12°C (Figure 4).

### 3.3 Potentially suitable distribution areas of *Colletotrichum acutatum* under current climate

**Figure 5:**
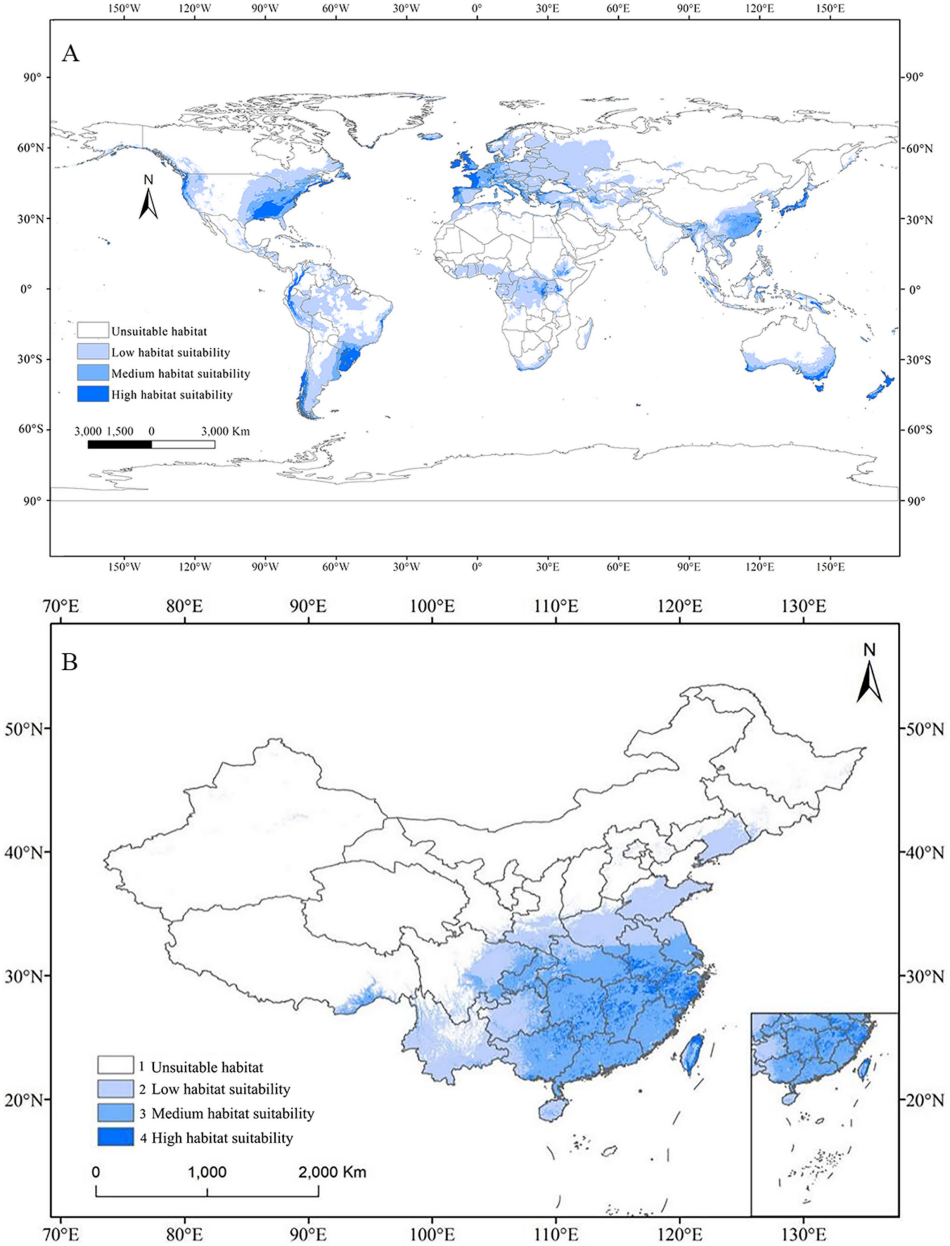
Potential distribution of *C. acutatum* under current climate scenario by MaxEnt. **(A)** Global habitat suitability map. **(B)** Potential habitat suitability map in China.

Currently, potential suitable habitats for this fungus have been divided into four grades: high suitability, medium suitability, low suitability, and unsuitable areas. The Figure 5 illustrated the global potential distribution of *C. acutatum* based on current environmental variables. The results indicated that, except for Antarctica, suitable habitats for *C. acutatum* were distributed across all other continents. The species’ primary habitats were located in Western Europe, the terrestrial regions of Oceania, Central Africa, coastal areas of South America, the southeastern coast of North America, and Eastern Asia. In Europe, areas suitable for *C. acutatum* were mainly found in southwestern Germany, the western coastal regions of the UK, most of the western coastal regions of France, and the southwestern coastal regions of Italy. In Oceania, suitable habitats were primarily distributed along the southern coast of Australia and in New Zealand. In South America, the species’ suitable habitats were mainly in southern Brazil, Uruguay, southern Chile, the western coastal regions of Peru, as well as northwestern Ecuador and Colombia. In North America, *C. acutatum* was predominantly found along the western and eastern coasts of the southeastern United States. In Asia, its distribution was mainly in southern China, Japan, areas bordering India and Myanmar, and coastal regions of Indonesia.

The total suitable habitat area was the sum of regions classified as highly suitable, moderately suitable, and lowly suitable. In Asia, the largest suitable habitat for *C. acutatum* was in China, covering 259.52 × 10^4^ km^2^ (Table 4). Specifically, the highly suitability area covered 16.60 × 10^4^ km^2^, the medium suitability area covered 121.77 × 10^4^ km^2^, and the low suitability area covered 121.16 × 10^4^ km^2^. The highly suitable areas were primarily distributed along the coastal regions south of 30°N, covering relatively small areas, including Zhejiang Province, northern Jiangxi Province, eastern coastal Fujian Province, southern Anhui Province, northeastern Hunan Province, southern coastal Guangdong Province, northeastern Taiwan, and other regions. The medium suitability area was larger and primarily situated south of the Qinling-Huaihe Line. This line in China represented the 800 mm annual precipitation line and the 0°C January isotherm.

**Table 4:** Prediction of suitable areas for *Colletotrichum acutatum* under current and future climatic conditions.

Decade scenarios	Predicted area (×10^4^ km^2^)					Comparison with current distribution (%)				
	Low habitat suitability	Medium habitat suitability	High habitat suitability	Unsuitable habitat	Total area	Low habitat suitability	Medium habitat suitability	High habitat suitability	Unsuitable habitat	Total area
Current	121.16	121.77	16.60	701.54	259.52					
2050s-SSP1-2.6	128.76	114.34	20.89	697.09	263.98	6.27	−6.10	25.86	−0.64	1.72
2050s-SSP3-7.0	129.70	114.78	26.66	689.93	271.13	7.05	−5.74	60.61	−1.66	4.47
2050s-SSP5-8.5	148.65	111.11	27.42	673.88	287.19	22.69	−8.75	65.22	−3.94	10.66
2070s-SSP1-2.6	100.91	110.14	28.28	721.75	239.32	−16.71	−9.55	70.37	2.88	−7.78
2070s-SSP3-7.0	142.24	105.74	15.07	698.02	263.05	17.40	−13.16	−9.23	−0.50	1.36
2070s-SSP5-8.5	151.80	112.11	23.95	673.20	287.87	25.29	−7.93	44.32	−4.04	10.92

### 3.4 Change in potentially suitable distribution areas of *Colletotrichum acutatum* under the future climate

**Figure 6:**
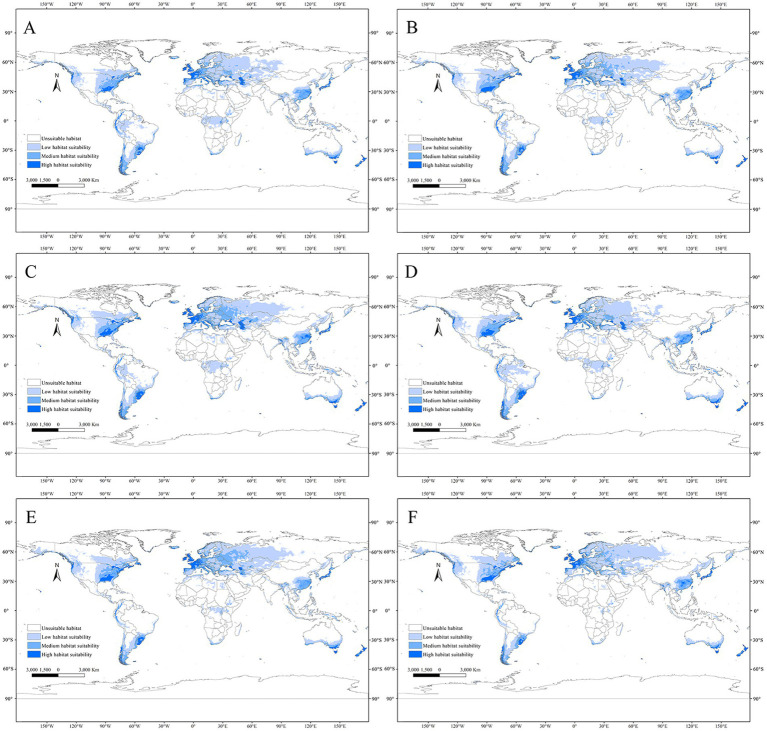
Global potential distribution map for *C. acutatum* under the future climate scenario. **(A)** ssp126 in the year 2050; **(B)** ssp370 in the year 2050; **(C)** ssp585 in the year 2050; **(D)** ssp126 in the year 2070; **(E)** ssp370 in the year 2070; **(F)** ssp585 in the year 2070.

In the future climate scenarios of SSP1-2.6, SSP3-7.0, and SSP5-8.5, the distribution of suitable habitats for *C. acutatum* in the 2050s and 2070s was predicted. Globally, the suitable habitat for *C. acutatum* was generally expanding inland. In North America, the suitable habitat in the southeastern United States was extending northwestward. In South America, low-suitability areas in the central part of the continent were becoming unsuitable, showing significant change. In Europe, the suitable habitat was gradually expanding towards the higher latitudes in the north. In Asia, the low-suitability areas in Russia were continuously expanding eastward (Figure 6).

**Figure 7:**
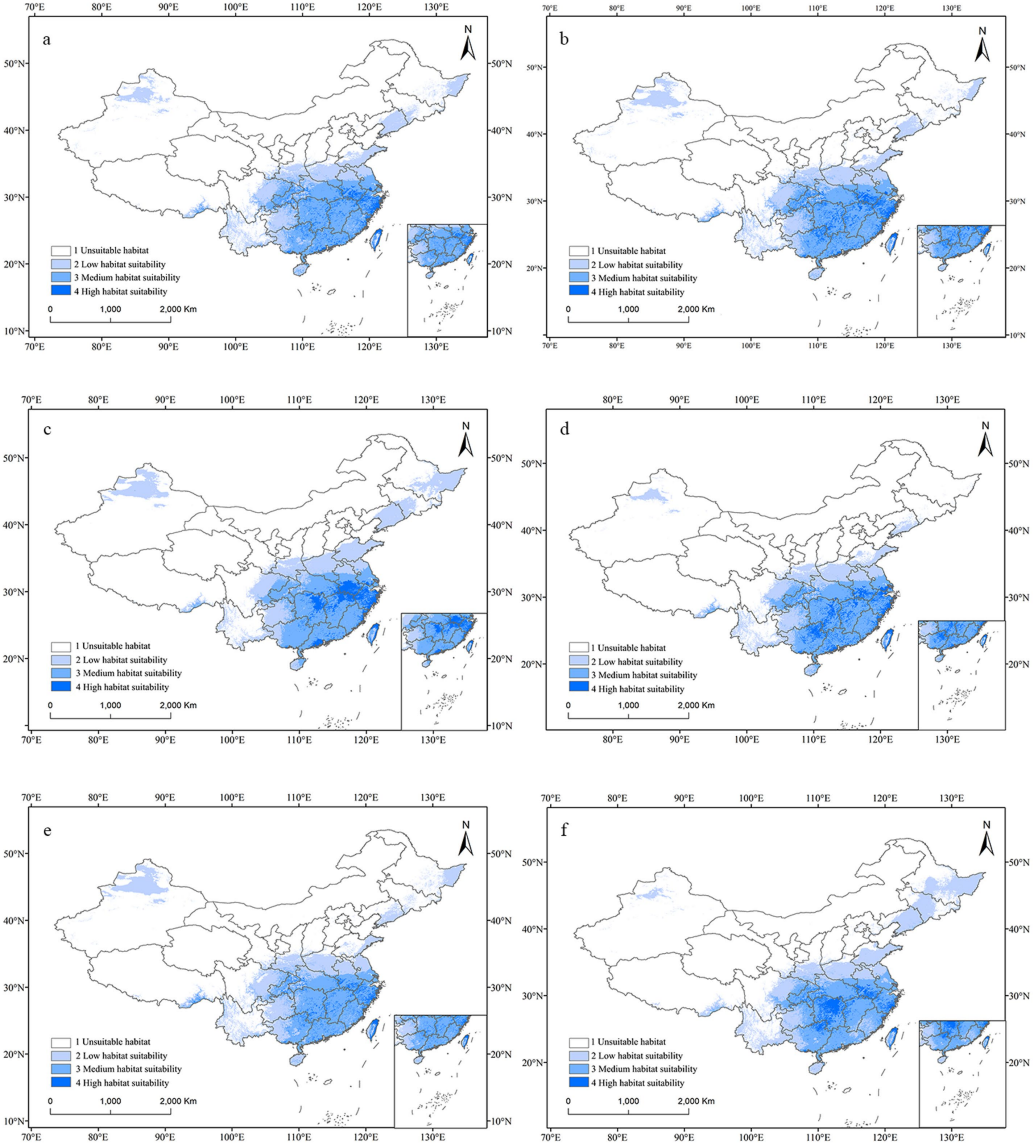
Potential distribution map for *C. acutatum* in China under the future climate scenario. **(A)** ssp126 in the year 2050; **(B)** ssp370 in the year 2050; **(C)** ssp585 in the year 2050; **(D)** ssp126 in the year 2070; **(E)** ssp370 in the year 2070; **(F)** ssp585 in the year 2070.

In China, under most future climate emission scenarios, the area of suitable habitats for *C. acutatum* was expected to increase (Figure 7). Only in the SSP1-2.6 scenario for the 2070s did the total suitable habitat area decrease, although the high suitability habitat area increased from 16.60 × 10^4^ km^2^ to 28.28 × 10^4^ km^2^ (Table 4). Under the SSP5-8.5 emission scenario, this species’ total suitable habitat area increased the most. In the 2050s, the total suitable habitat area expanded to 287.19 × 10^4^ km^2^, an increase of 10.66%. The high suitability habitat area and low suitability habitat area also increased significantly, by 65.22 and 22.69%, respectively. By the 2070s, the total suitable habitat area had expanded by 10.92%, with the high suitability habitat area expanding by 44.32%.

In future climate scenarios, *C. acutatum* showed a trend of shifting towards higher latitudes in northern regions. From the map, it can be observed that the Sanjiang Plain and the Junggar Basin will transition from unsuitable areas to low-suitability areas. The high suitability habitat for this species expanded from the southeast coastal areas inland. Northeastern Jiangxi Province, northeastern Hunan Province, southern Anhui Province, southern Guangdong Province, southeastern Guangxi Zhuang Autonomous Region, and western Zhejiang Province became high suitability habitats for *C. acutatum*. Inner Mongolia Autonomous Region, Qinghai Province, Hebei Province, Ningxia Hui Autonomous Region, Tianjin Municipality, and Beijing Municipality remained unsuitable areas for this species.

### 3.5 Centroid changes in potential distribution

**Figure 8:**
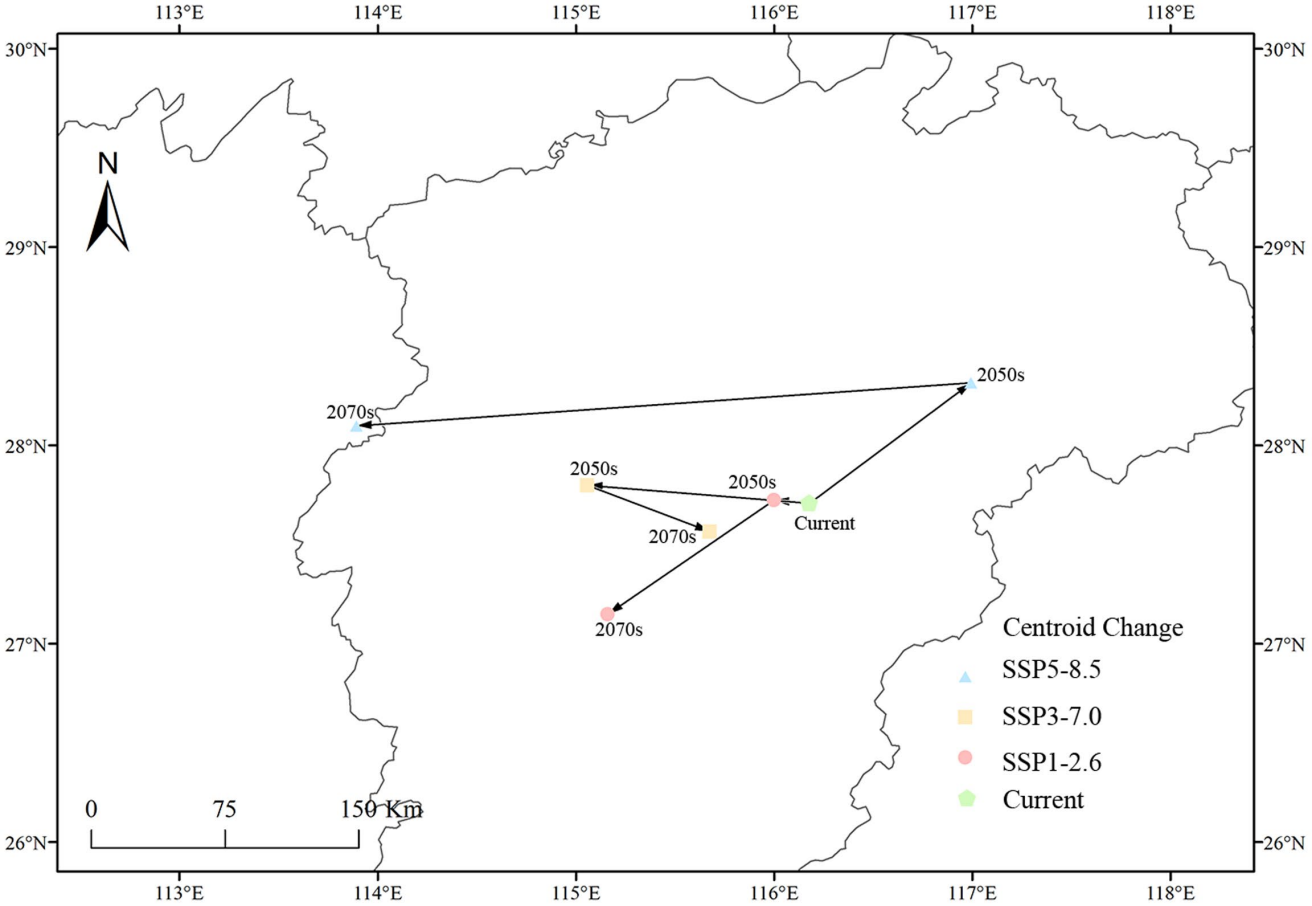
Changes in the centroid of the potential highly suitable distribution of *C. acutatum* in China.

In this study, the movement of the distribution centroid of the high suitability habitat of *C. acutatum* under different climate scenarios was illustrated (Figure 8). Currently, under present conditions, the distribution centroid of the high suitability habitat for *C. acutatum* was located in Chongren County, Fuzhou City, Jiangxi Province (116°10′46″E, 27°42′33″N). In future scenarios, the centroid of this high suitability habitat generally shifted southwestward, indicating the gradual inland expansion of *C. acutatum*’s distribution in China. Under SSP1-2.6 and SSP3-7.0 scenarios, the centroid of the high suitability habitat for *C. acutatum* initially shifted northwestward. Interestingly, for these two emission scenarios, there was a segment of the centroid’s shifting trajectory that overlaps. In the SSP1-2.6 scenario, by the 2070s, the centroid shifted southwestward and reaches Jishui County, Ji’an City, Jiangxi Province (115°9′42″E, 27°8′56″N). Under the SSP5-8.5 scenario, the centroid of the high suitability habitat for *C. acutatum* showed the greatest degree of displacement. Initially, it shifted northeastward to Guixi City, Yingtan City, Jiangxi Province (116°10′46″E, 27°42′33″N), and then southwestward to Liuyang City, Changsha City, Hunan Province (113°53′40″E, 28°5′58″N).

## 4 Discussion

Species distribution modeling is a model tool based on species occurrence data and environmental variables to predict the potential geographic distribution of species under specific spatiotemporal conditions. It is a hot topic in current biological research. The relationship between species distribution and the environment is intricate and complex (Zhao et al., 2022). For different research objectives, ensuring the accuracy of results requires selecting appropriate models and parameters. The main goal of this study is to predict the current and future potential distribution of a species using known species occurrence points.

In recent years, the MaxEnt model has gained popularity as a robust ecological niche model capable of accurately predicting species distributions. When predicting the range of species distributions, this model assumes that species exist in all areas where environmental conditions are suitable and do not exist in unsuitable areas (Li et al., 2020). The research indicates that under known conditions, the larger the entropy of a species, the closer the predicted results are to reality (Steven et al., 2006). The default parameters of MaxEnt were initially derived by developers through testing known species distribution points and distribution areas (Steven and Miroslav, 2008). Researchers test optimal model parameters through extensive species distribution data and experimental designs to simplify the settings of the MaxEnt model to the greatest extent possible. The accuracy of MaxEnt’s default parameters can be evaluated by comparing the predicted results obtained through software computations with actual observations (Hirzel et al., 2006). However, when used to predict future species distribution ranges, these results cannot be verified. Moreover, using only default parameters for computation can lead to overfitting and reduce the accuracy of the model results (Aleksandar and Robert, 2014). Therefore, when using the MaxEnt model to predict species’ future distributions, it is necessary to reduce the model’s omission rate to improve prediction accuracy (Muscarella et al., 2014). In this study, based on occurrence data and environmental variables of *C. acutatum*, the parameters of the MaxEnt model were adjusted. After adjustment, the model’s omission rate was 0.073%, which is less than 5%, and the delta AICc was 0. These adjusted parameters are suitable for predicting the current and future distribution of *C. acutatum*.

The predictions of the MaxEnt model depend not only on its parameter settings but also on the selection of species distribution and environmental factors. Therefore, to ensure the accuracy of the MaxEnt model input data, species distribution data are collected as extensively as possible. This helps to avoid sample bias caused by insufficient species occurrence points. Generally, the selected species distribution points should adequately cover the species’ distribution range while avoiding biases caused by overfitting (Wisz et al., 2008). Therefore, in this study, the distribution data of this species were filtered to retain only one distribution point per grid. Using ArcGIS software, the distribution range of these points was validated, and only the valid distribution points were retained. Secondly, environmental factors were selected using VIF, Pearson correlation coefficients, and contribution rates, resulting in nine environmental factors being included in the MaxEnt model construction. In this research, AICc values were used to select the optimal combination of feature types and regularization multipliers, thereby reducing the model’s complexity. AUC is an important model evaluation metric in machine learning, widely used to assess the accuracy of MaxEnt model results. The AUC value in this MaxEnt model was 0.95, indicating reliable predictive results. Huercha et al. used the MaxEnt model to predict the distribution of *Dermacentor marginatus* and found the prediction results to be reliable (Huercha et al., 2020). In this study, relying on bioclimatic variables, the current and future potential distribution of *C. acutatum* was predicted. This research identifies potential risk areas for future invasion of *C. acutatum* using the MaxEnt model in the 2050s and 2070s.

*C. acutatum* is a globally widespread plant pathogenic fungus. It is primarily distributed in coastal areas between 20°N-70°N and 30°S-60°S. These regions are mainly characterized by temperate oceanic climates, subtropical monsoons, humid subtropical climates, and temperate continental climates. The abundant rainfall and relatively high temperatures in these areas create favorable conditions for the reproduction and survival of *C. acutatum*. The main regions in China where anthracnose disease occurs are concentrated in the middle and lower reaches of the Yangtze River Plain and the southeastern hills, which is consistent with the predictions of this study (Huanhuan, 2023). Our results indicated that precipitation played a crucial role in forming suitable habitats for *C. acutatum*, and the mean temperature of the driest quarter was also an important influencing factor. In China, the Qinling-Huaihe Line is a geographical boundary in China that distinguishes the northern and southern regions. There are significant differences in climate characteristics, geographical landscapes, and other aspects on both sides of this line. This line separates the humid and semi-humid areas, temperate and subtropical zones, and subtropical monsoon climate and temperate monsoon climate. In winter, south of the Qinling-Huaihe Line is mild and less rainy, while north of the line is cold and dry (Sunan and Chengyuan, 2024). According to the predictions, the suitable habitat of *C. acutatum* was distributed south of the Qinling-Huaihe Line, where the annual precipitation exceeds 800 mm, winter temperatures are relatively high, and there is minimal freezing. Research indicated that rainfall contributed to the reproduction and spread of *C. acutatum*, increasing both the infection rate and the amount of infection in plants (Mckay et al., 2014). Therefore, this MaxEnt model accurately predicted the environmental factors that affect the survival of *C. acutatum*.

This study demonstrated that future climate change conditions favored the survival of *C. acutatum*. The spread of *C. acutatum* in its distribution areas will pose extensive risks to crops and fruit trees. This study predicted the distribution of *C. acutatum* under three socioeconomic pathways until the 2070s, providing more scenarios than previous research. In China, *C. acutatum* was mainly distributed in southeastern coastal regions such as Zhejiang, Jiangxi, Guangdong, Taiwan, Anhui, and others. These provinces are characterized by flat terrain, proximity to the ocean, high summer temperatures with humidity, significant precipitation, and short duration of low winter temperatures, which meet the environmental requirements for *C. acutatum* habitat. Under future environmental conditions, there was a trend for *C. acutatum* habitat to shift inland in China, with the greatest movement observed under the SSP5-8.5 scenario. This indicated a significant impact of high carbon emissions on *C. acutatum*. Within a certain range, higher future carbon dioxide emissions will increasingly favor the survival of this species. Studies have shown that as the global climate warms, the atmosphere’s ability to hold water increases, leading to a continued strengthening of the global water cycle, primarily expressed in the increase in total precipitation and the intensification of precipitation extremes (Zhang et al., 2021). These extreme weather changes will lead to an increase in plant disease rates and facilitate the spread of *C. acutatum*.

The southern regions of China are generally suitable for the survival of *C. acutatum*, so enhancing its prevention and control is of utmost importance. First and foremost, it is crucial to strengthen the prevention and control system, increase publicity on the damage caused by anthracnose and control measures, raise awareness among relevant personnel, and promptly detect and treat the disease. Secondly, optimize cultivation management by selecting suitable sites and trees, and timely prune dead branches and leaves to maintain good ventilation between plants (de los Santos García de Paredes and Romero Muñoz, 2002). Choosing disease-resistant varieties can help reduce the risk of fungal infection to some extent. Upon discovering *C. acutatum* infection in plants, immediately prune and burn the infected parts to prevent the spread and dissemination of the pathogen. Lastly, when the disease becomes severe, chemical control is often effective. When using chemical control methods, it is advisable to alternate the use of fungicidal pesticides to prevent the development of resistance in the pathogens (Peres et al., 2002).

## 5 Conclusion

In summary, globally, under future climate conditions, the suitable habitat for *C. acutatum* expanded further inland, with rainfall and temperature being the main environmental factors limiting its distribution. In China, according to the prediction results, areas south of the Qinling Mountains and east of the Huai River were likely to become suitable habitats for this fungus. Given its wide distribution and ease of spread, *C. acutatum* poses significant challenges to the control of anthracnose. Therefore, relevant authorities should increase their attention to the prevention and control of *C. acutatum*, enhance monitoring efforts, and implement measures to mitigate its impact on agricultural development.

## Data Availability

The datasets presented in this study can be found in online repositories. The names of the repository/repositories and accession number(s) can be found at: https://doi.org/10.6084/m9.figshare.26175430.v1 and https://doi.org/10.15468/dl.6a554m (GBIF).

## Footnotes

^1^https://www.worldclim.org
